# Prenatal Stress Exposure Related to Maternal Bereavement and Risk of Childhood Overweight

**DOI:** 10.1371/journal.pone.0011896

**Published:** 2010-07-30

**Authors:** Jiong Li, Jørn Olsen, Mogens Vestergaard, Carsten Obel, Jennifer L. Baker, Thorkild I. A. Sørensen

**Affiliations:** 1 Department of Epidemiology, School of Public Health, University of Aarhus, Aarhus, Denmark; 2 Department of Epidemiology, School of Public Health, University of California Los Angeles, Los Angeles, California, United States of America; 3 Research Unit for General Practice, University of Aarhus, Aarhus, Denmark; 4 Department of General Practice, School of Public Health, University of Aarhus, Aarhus, Denmark; 5 Institute of Preventive Medicine, Copenhagen University Hospital, Copenhagen, Denmark; University of Las Palmas de Gran Canaria, Spain

## Abstract

**Background:**

It has been suggested that prenatal stress contributes to the risk of obesity later in life. In a population–based cohort study, we examined whether prenatal stress related to maternal bereavement during pregnancy was associated with the risk of overweight in offspring during school age.

**Methodology/Principal Findings:**

We followed 65,212 children born in Denmark from 1970–1989 who underwent health examinations from 7 to 13 years of age in public or private schools in Copenhagen. We identified 459 children as exposed to prenatal stress, defined by being born to mothers who were bereaved by death of a close family member from one year before pregnancy until birth of the child. We compared the prevalence of overweight between the exposed and the unexposed. Body mass index (BMI) values and prevalence of overweight were higher in the exposed children, but not significantly so until from 10 years of age and onwards, as compared with the unexposed children. For example, the adjusted odds ratio (OR) for overweight was 1.68 (95% confidence interval [CI] 1.08–2.61) at 12 years of age and 1.63 (95% CI 1.00–2.61) at 13 years of age. The highest ORs were observed when the death occurred in the period from 6 to 0 month before pregnancy (OR 3.31, 95% CI 1.71–6.42 at age 12, and OR 2.31, 95% CI 1.08–4.97 at age 13).

**Conclusions/Significance:**

Our results suggest that severe pre-pregnancy stress is associated with an increased risk of overweight in the offspring in later childhood.

## Introduction

Obesity is of major public health concern [1] and recent research has mainly been devoted to the gene-environment interactions in the evolving epidemic. [2], [3] Although life styles and variations in genotype are important determinants, they cannot fully explain the etiology of obesity.[1], [4] Mounting evidence indicates that exposures during fetal life also play a important role.[5], [6] Those processes may not cause obesity directly but rather alter an individual's susceptibility to develop obesity in a given obesogenic environment and genetic background. [7], [8]

Experimental studies have shown that prenatal stress leads an organism to forecast an adverse future environment and to change its developmental trajectory accordingly.[8], [9] Exposure to excess stress hormones during fetal life is associated with a number of physiological pathways that can be linked to future obesity [7] via glucocorticoid programming. [10], [11] It remains to be elucidated whether such mechanisms operate in humans. [8]

We hypothesized that prenatal stress exposure leads to an increased susceptibility to obesity in childhood. [7], [8] It is well recognized that the adverse impacts of stress on health in modern society has been increasing. [12] However, stress is difficult to measure in research. [13] We thus used exposure to bereavement to obtain a large stress contrast between the exposed and the unexposed. Bereavement due to the death of a close relative is classified as one of the most stressful life events. [14] It conceivably affects almost all and induces excess stress hormones, regardless of their coping styles. [15] In this population-based cohort study, we used maternal bereavement during prenatal life as an indicator of stress, to examine whether stress during fetal life was associated with childhood overweight in the offspring at different school ages.

## Methods

### Study design, participants, exposure, and outcomes

We conducted a population-based follow up study based on Danish national registers. All live born children and new residents in Denmark are assigned a unique civil personal registration (CPR) number, allowing accurate linkage of data between registries. [16]Within this system, it is possible to track any particular person over decades and link data at an individual level on demographic, vital status, social and economic conditions, and health information in a single study. [16]

We first identified all children born in Denmark from 1970 to1989 through the Danish Civil Registration System and obtained their date of birth and gestational age. [17] Using their personal identification number, these children were then linked to their parents, siblings, grandparents, and mother's siblings, for whom information on the date and cause of death could be retrieved. We thus identified children born to mothers who experienced the death of a close relative (partner/spouse, child, sibling, or parent) during the prenatal time period (12 months prior to pregnancy until the birth of the index child). These children were included in the exposed cohort and the remaining children comprised the unexposed cohort. Exposed children were further categorized into sub-groups by timing of bereavement: 12 to 7 months before pregnancy, 6 to 0 months before pregnancy, and during pregnancy. The children were followed to the end of 2004 with minimal loss to follow up (less than 0.03 during 25 years of follow up related to disappearance from registers).[18] Children were further linked to the Copenhagen School Health Record Registry (CSHRR), [19] which has data on mandatory health examinations of children enrolled in public or private schools in the municipality of Copenhagen. During the examination, school doctors or nurses measured the children, and each child was assigned a health card in which yearly height and weight measurements were recorded. We included all 65,212 singleton children as subjects in this study.

The definition of childhood overweight at specific ages (7, 8, 9, 10, 11, 12, and 13) was based on an international gender- and age-specific BMI reference.[20] The measurements of weight and height were retrieved from the CSHRR. [19] Body mass index (BMI) was calculated as weight (kg) / height (m)^2^. As the height and weight measurements were collected as part of routine school health examinations, there was not a quality control program, per se, in place. The doctors and nurses, however, recorded the heights and weights with a great attention to detail; heights were recorded to the nearest half-centimeter and weights were recorded to the nearest 100 g. When the data were computerized, the data entry program applied a series of range checks, and this precluded typographical errors. Furthermore, as we had longitudinal measurements of the children, we plotted the growth curves for the children and examined them for unusual values. Since the records are in the municipal archive, we were able to verify these values. [19]

Information on demographic, vital statistics, and family relationships was retrieved from the Danish Civil Registration System, which was established in 1968. [21] Information on birth outcomes, such as birth weight and gestational age, were obtained from the Danish Medical Birth Register, [17] which was established in 1973. [19] Information on socio-economic factors (maternal age, maternal residential place, maternal education, maternal income, and maternal cohabitation status) was obtained from the Integrated Database for Longitudinal Labour Market Research (IDA), but these data were only available from 1980 and onwards. [22]

### Statistical analysis

The data were analysed in SAS (version 9.1). The differences in mean BMI values between the exposed and unexposed groups at the ages of 7, 8, 9, 10, 11, 12, and 13 years were analysed with linear regression. Chi-square (χ^2^) test was used to test the association between exposure and outcomes of overweight and obesity at a specific age. Odds ratios (ORs) for overweight at the different ages between the exposed and unexposed groups were calculated using logistic regression. We adjusted for the following potentially confounding variables or intermediate variables: gender (male, female), birth year (1970–1978, 1979–1983, 1983–1989), gestational age (<37 weeks, > = 37 weeks, unknown), birth weight (<3000 g, 3000–3350 g, 3350–3700, >3700 g, unknown), and maternal age (<27 years, 27–30 years, 31 years and over), maternal school education (0 to 9 years, 10 to 11 years, 12+ years), maternal income (lowest quartile, 2^nd^ quartile, 3^rd^ quartile, highest quartile in the calendar year), and maternal cohabitation status (yes, no).

Sensitivity to prenatal exposure to stress is expected to vary with different developmental time windows and by gender. [23] We therefore performed stratified analyses according to different timing of exposure and gender.

### Ethics

This study was conducted according to the principles expressed in the Declaration of Helsinki. The Danish Data Protection Agency approved the study (J.nr. 2008-41-2555, J.nr.2008-41-2680). The study was based on secondary data and no individuals were approached, nor did we have access to any other information from the participants. Thus it is not necessary to have the written consent.

## Results

Among the 65,212 children, 459 were exposed to maternal bereavement during the prenatal period. The baseline characteristics of the exposed and unexposed children were comparable, although exposed children were more often born preterm or born during later years, mainly due to better options for identification of loss of grandparents (Table S1).

**Figure 1:**
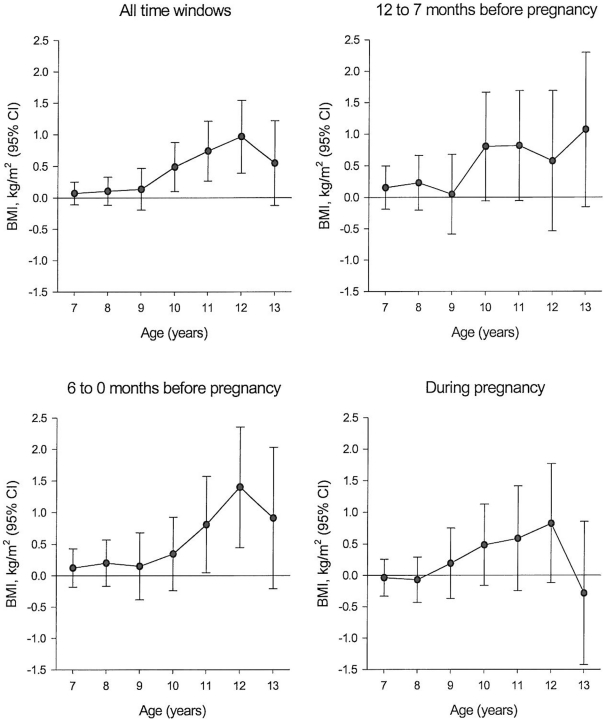
Mean BMI (kg/m^2^) differences between the exposed and unexposed children at 7 to 13 years of age by timing of exposure (linear regression).

From 10 years of age and onwards, exposed children had a higher mean BMI than unexposed children (mean difference 0.48, 95% confidence interval [CI] 0.10–0.87 at 10 years; 0.74, 95% CI 0.26–1.31 at 11 years; 0.96, 95% CI 0.39–1.54 at 12 years, and 0.54, 95% CI 0.13–1.22 at 13 years). Similar results were observed when stratifying the results according to the time period of bereavement (12–7 months before pregnancy, 6–0 months before pregnancy, and during pregnancy) (Figure 1).

Table 1 presents the prevalence of overweight and obesity by age. Compared to unexposed children, exposed children had higher rates of overweight at all ages. The differences were, however, statistically significant only between the ages of 10 and 13 years (P<0.001). Similar results were obtained for obesity, but the numbers were too low for a thorough analysis.

**Table 1:** Prevalence of Overweight and Obesity by Status of Exposure and Age.*

Age (years)	Exposed			Unexposed		
		Overweight	Obesity		Overweight	Obesity
	N	n (%)	n (%)	N	n (%)	n (%)
**7**	326	37 (11.4)	6 (1.8)	45305	4693 (10.4)	721 (1.6)
**8**	279	32 (11.5)	6 (2.1)	39053	4107 (10.5)	692 (1.8)
**9**	173	24 (13.9)	4 (2.3)	28022	3619 (12.9)	470 (1.7)
**10**	164	35 (21.3)	4 (2.4)	24522	3404 (14.4)	527 (2.1)
**11**	133	32(24.1)	3 (2.2)	21499	3100 (14.3)	459 (2.1)
**12**	105	28(26.7)	4 (3.8)	18144	2615 (14.4)	397 (2.2)
**13**	87	22(25.3)	2 (2.3)	14531	2293 (15.8)	340 (2.3)

*Chi-square (χ^2^) test for association between exposure and outcomes of overweight and obesity at a specific age: 7 years: P = 0.6051; 8 years: P = 0.6502; 9 years: P = 0.7080; 10 years: P = 0.0060; 11 years: P = 0.0016; 12 years: P = 0.0004; 13 years: P = 0.0154.

**Figure 2:**
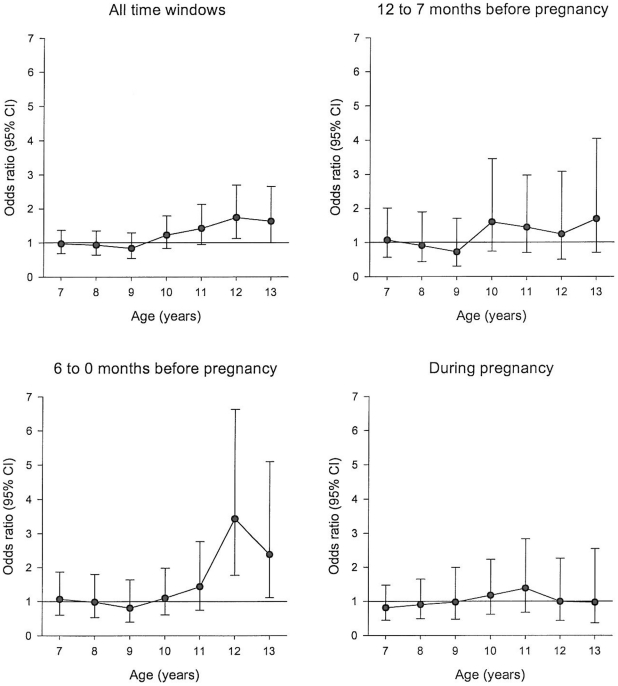
Odds ratio of overweight at ages 7 to 13 years between exposed and unexposed children by timing of exposure (logistic regression*). *Odds ratios adjusted for birth year, birth weight, gestational age, sex and maternal factors (age, education, income, and cohabitation status).

Figure 2 shows the odds ratios (ORs) for overweight between exposed and unexposed groups, adjusted for several covariates. The odds of being overweight were higher among the exposed from 10 years of age and onwards. For example, the OR at 12 years of age was 1.68 (95%CI 1.08–2.61) and 1.63 at 13 years of age (95%CI 1.00–2.61). The highest ORs were observed for exposure during the period of 6–0 months prior to pregnancy (OR 3.31, 95%CI 1.71–6.42 at age 12, OR, 2.31, 95%CI 1.08–4.97 at age 13).

## Discussion

In this population-based cohort study, children exposed to severe prenatal stress had higher BMI values and a higher prevalence of overweight when they approached the age of 10 years. The association was not significantly modified by gender, birth year, birth weight, gestational age, and maternal factors (age, education, income, and cohabitation status). The association was particularly strong when the exposure happened in the months just before conception.

Observational studies have suggested that the intrauterine life may be a critical period for the development of obesity later in life. [6] Children born to women who were exposed to the 1944–45 Dutch famine had an increased risk of obesity later in life.[24] Although this finding was attributed to lack of energy or nutrition to the fetus during the siege, a programming effect of prenatal stress is a plausible alterative explanation. Fetal growth variables like birth weight were often used to estimate the role of prenatal exposures on the future risk of obesity but results have not been conclusive, [25]–[27] These growth variables are convenient markers or surrogates for summing the interaction between the fetal environment and genetic influences, and their effects may often be subject to the interaction with other early risk factors, or the selection of different outcomes in childhood.[26], [27] Prenatal factors like maternal gestational diabetes, [28] maternal smoking during pregnancy,[29] and malnutrition [30] have also been associated with obesity in later life. However, the inconsistent associations and variations in the magnitude of these estimates call for more investigations on the upstream causes of obesity during prenatal period.

Findings from experimental studies support the biological plausibility of the link between prenatal stress and obesity later in life. [10], [11] These studies suggest that glucocorticoid, one of the stress hormones, has a programming effect on obesity. [10], [11] Excess maternal glucocorticoid can enter the fetal circulation, which may cause endocrine dysregulation and influence the development of the hypothalamic-pituitary-adrenal (HPA) axis, as well as the metabolism later in life. [10], [11] This programming may consequently affect the individuals metabolism by promoting the conversion of proteins and lipids to usable carbohydrates, increase food-seeking behaviors, and inhibit insulin action on glucose uptake. [10], [11] It has also been shown that exogenous glucocorticoids in pregnant rats causes insulin resistance in the offspring, as well as epigenetic changes in the developing brain, [31] which may lead to an increased susceptibility to obesity. The excessive maternal glucocorticiods in bereaved mothers [15] may have such a direct fetal programming effect on obesity in the offspring, and maybe a plausible explanation for the findings we presented here. Additionally, bereavement often leads to more adverse behaviors like smoking and alcohol consumption in mothers, which may also contribute to the observed associations. [6]

It is interesting to observe that the risk of overweight did not emerge until the pre-pubertal age. Preliminary evidence, mainly from animal studies, has suggested that environmental pollutants during fetal life may cause the puberty to occur at a younger age.[32] Some pointed to differential endocrine regulatory mechanisms linked to pubertal development acting in the perinatal and the pre-pubertal period. [32], [33]Evidence from epidemiological studies also suggests that the prenatal period may represent an early window of susceptibility to long-term ‘programming’ of puberty development. [34] For example, an abnormal prenatal environment of children born SGA, may alter the endocrine status and the sensitivity of the receptors for endocrine and metabolic signaling, which may affect maturation of brain and gonads. [34] The biological explanation underlying this time-specific association remains unknown but prenatal stress may affect both the timing or the velocity of the pubertal growth spurt [32] and the risk of future obesity,[5] which may be closely linked. [35]

One of the important findings was related to the timing of exposure where the months just before conception seem to be the most susceptible period. Previous studies have suggested that women become less sensitive to stress as pregnancy advances. [36], [37] The reactivity of the two major components involved in the maternal stress response, the HPA and sympathetic-adrenal-medullary axis, are dampened during pregnancy.[36], [37] For example, the known decrease in vulnerability to acute stress induced by earthquake may reflect increasing protection of the mother and fetus from stress or other adverse influences during pregnancy. [38]The Dutch Famine study found that exposure to maternal malnutrition during mid-pregnancy was associated with childhood obesity. [24] Bereavement may lead to a similar pattern of excess stress hormones during the initial phase, as do other acute stressors like earthquake. It also causes long-term interruptions in the hormonal balance, leading to an allostatic load, [39]which implies a high level of stress hormones in mothers. Thus the event of bereavement before pregnancy could lead to excessive glucocorticoids during early pregnancy. This may explain the higher ORs associated with pre-pregnancy stress in this study. Unfortunately, our study did not have enough statistical power to further differentiate the effect of the exposure in more narrow time windows around gestation.

The strengths of the study are its population-based longitudinal design, objective measurement of exposure, and the high quality data on endpoints that are recorded independent of the exposure. The study included virtually all school children in the Copenhagen municipality area, and selection bias is unlikely. The CSHRR is a unique data source for obesity research with detailed data on growth with a high validity. [19] Information on exposure, death of relatives, is accurately recorded in Danish registers. [21]

One limitation of the study is the classification of overweight based on BMI values, as they might not be an optimal marker for body fatness in childhood due to the fact that BMI estimates not only the fat tissue, but also fat free mass tissue. [26] Other limitations of the study include the relatively small size of the exposed cohort, lack of biomarkers of cortisol exposure, and lack of information about socio-economic status during the period of 1970–1979. Denmark is an affluent society with a comprehensive public health system that provides equal access to health care to all, independent of socio-economic status, and mortality rates are low among children and young adults.We also lacked data on lifestyles. However, bereavement may also lead to more adverse life styles that lie in the pathways between exposure and the outcome, thus these life styles should not necessarily be controlled for in the analyses.[40]

Prenatal stress, like maternal bereavement during pregnancy, is expected to affect the fetus and increase the frequency of adverse birth outcomes. [41] These factors may be in the pathways between prenatal stress and overweight and it can be argued that they should not be adjusted for in the analyses. Conversely, these factors also have many other causes that could reflect a lack of comparability at baseline in our study. Nevertheless, our results indicate that preterm birth or low birth weight are not strong intermediates.

In conclusion, our findings suggest that severe pre-pregnancy stress may increase the susceptibility to overweight in childhood. Overweight and obesity in childhood are associated with adult obesity and many other negative health consequences. [42], [43] In this study, we have focused upon one of the most severe and rare stress exposure. It is likely that many women with poor coping mechanisms may reach similar hormonal responses at lower levels of stress, which is more prevalent at the population level. [39]. This should be taken into consideration for prevention strategies, especially in the light that recent experimental animal research has shown that those aberrant phenotypes induced in utero can be reversed. [44]

## Supporting Information

Table S1 Baseline characteristics of the study population.*
(0.10 MB DOC)

## References

[1] Haslam DW, James WP (2005). Obesity.. The Lancet.

[2] Cecil JE, Tavendale R, Watt P, Hetherington MM, Palmer CN (2008). An obesity-associated FTO gene variant and increased energy intake in children.. N Engl J Med.

[3] Hetherington MM, Cecil JE (2010). Gene-Environment Interactions in Obesity.. Forum Nutr.

[4] Sørensen TIA (2009). Challenges in the study of causation of obesity.. Proc Nutr Soc.

[5] Heindel JJ, vom Saal FS (2009). Role of nutrition and environmental endocrine disrupting chemicals during the perinatal period on the aetiology of obesity.. Mol Cell Endocrinol.

[6] Huang JS, Lee TA, Lu MC (2007). Prenatal Programming of Childhood Overweight and Obesity.. Matern Child Health J.

[7] Gluckman PD, Hanson MA (2008). Developmental and epigenetic pathways to obesity: an evolutionary-developmental perspective.. Int J Obes.

[8] Gluckman PD, Hanson MA, Cooper C, Thornburg KL (2008). Effect of In Utero and Early-Life Conditions on Adult Health and Disease.. N Engl J Med.

[9] Barker DJ (2007). The origins of the developmental origins theory.. J Intern Med.

[10] Bouret SG (2009). Early Life Origins of Obesity: Role of Hypothalamic Programming. [Review].. J Pediatr Gastroenterol Nutr.

[11] Drake AJ, Tang JI, Nyirenda MJ (2007). Mechanisms underlying the role of glucocorticoids in the early life programming of adult disease.. Clin Sci.

[12] The American Institute of Stress (2009). http://www.stress.org/americas.htm.

[13] Monroe SM, Roberts JE (1990). Conceptualizing and measuring life stress: problems, principles, procedures, progress.. Stress Med.

[14] Skodol AE, Shrout PE (1989). Use of DSM-III axis IV in clinical practice: rating etiologically significant stressors.. Am J Psychiatry.

[15] Goodkin K, Baldewicz TT, Blaney NT, Asthana D, Kumar M, Stroebe MS, Hansson RO, Stroebe W, Schut H (2001). Physiological effects of bereavement and bereavement support group interventions.. Handbook of bereavement research.

[16] Frank L (2000). Epidemiology. When an entire country is a cohort.. Science.

[17] Knudsen LB, Olsen J (1998). The Danish Medical Birth Registry.. Dan Med Bull.

[18] Li J, Vestergaard M, Obel C, Precht DH, Christensen J (2009). Prenatal stress and cerebral palsy: a nationwide cohort study in Denmark.. Psychosom Med.

[19] Baker JL, Olsen LW, Andersen I, Pearson S, Hansen B (2009). Cohort Profile: The Copenhagen School Health Records Register.. Int J Epidemiol.

[20] Cole TJ, Bellizzi MC, Flegal KM, Dietz WH (2000). Establishing a standard definition for child overweight and obesity worldwide: international survey.. BMJ.

[21] Pedersen CB, Gotzsche H, Moller JO, Mortensen PB (2006). The Danish Civil Registration System. A cohort of eight million persons.. Dan Med Bull.

[22] Denmark Statistics (1991). http://www.dst.dk/.

[23] Mueller BR, Bale TL (2006). Impact of prenatal stress on long term body weight is dependent on timing and maternal sensitivity.. Physiol Behav.

[24] Ravelli GP, Stein ZA, Susser MW (1976). Obesity in young men after famine exposure in utero and early infancy.. N Engl J Med.

[25] Parsons TJ, Power C, Logan S, Summerbell CD (1999). Childhood predictors of adult obesity: a systematic review.. Int J Obes Relat Metab Disord.

[26] Labayen I, Moreno LA, Blay MG, Blay VA, Mesana MI (2006). Early Programming of Body Composition and Fat Distribution in Adolescents.. J Nutr.

[27] Singhal A, Wells J, Cole TJ, Fewtrell M, Lucas A (2003). Programming of lean body mass: a link between birth weight, obesity, and cardiovascular disease?. Am J Clin Nutr.

[28] Gillman MW, Rifas-Shiman S, Berkey CS, Field AE, Colditz GA (2003). Maternal Gestational Diabetes, Birth Weight, and Adolescent Obesity.. Pediatrics.

[29] Sharma AJ, Cogswell ME, Li R (2008). Dose-Response Associations Between Maternal Smoking During Pregnancy and Subsequent Childhood Obesity: Effect Modification by Maternal Race/Ethnicity in a Low-Income US Cohort.. Am J Epidemiol.

[30] Philipsen NM, Philipsen NC (2008). Childhood Overweight: Prevention Strategies for Parents.. J Perinat Educ.

[31] Welberg LAM, Seckl JR, Holmes MC (2001). Prenatal glucocorticoid programming of brain corticosteroid receptors and corticotrophin-releasing hormone: possible implications for behaviour.. Neuroscience.

[32] Roy JR, Chakraborty S, Chakraborty TR (2009). Estrogen-like endocrine disrupting chemicals affecting puberty in humans—a review.. Med Sci Monit.

[33] Jacobson-Dickman E, Lee MM (2009). The influence of endocrine disruptors on pubertal timing.. Curr Opin Endocrinol Diabetes Obes.

[34] Schoeters G, Den HE, Dhooge W, van LN, Leijs M (2008). Endocrine disruptors and abnormalities of pubertal development.. Basic Clin Pharmacol Toxicol.

[35] Aksglaede L, Juul A, Olsen LW, Sørensen TIA (2009). Age at puberty and the emerging obesity epidemic.. PLoS One 2009;.

[36] Matthews KA, Rodin J (1992). Pregnancy alters blood pressure responses to psychological and physical challenge.. Psychophysiology.

[37] Schulte HM, Weisner D, Allolio B (1990). The corticotrophin releasing hormone test in late pregnancy: lack of adrenocorticotrophin and cortisol response.. Clin Endocrinol (Oxf).

[38] Glynn LM, Wadhwa PD, Dunkel-Schetter C, Chicz-DeMet A, Sandman CA (2001). When stress happens matters: Effects of earthquake timing on stress responsivity in pregnancy.. Am J Obstet Gynecol.

[39] McEwen BS (1998). Protective and damaging effects of stress mediators.. New Engl J Med.

[40] Rothman KJ, Greenland S, Lash TL (2008). Modern Epidemiology. Third ed..

[41] Khashan AS, McNamee R, Abel KM, Mortensen PB, Kenny LC (2009). Rates of preterm birth following antenatal maternal exposure to severe life events: a population-based cohort study.. Hum Reprod.

[42] Baker JL, Olsen LW, Sørensen TIA (2007). Childhood Body-Mass Index and the Risk of Coronary Heart Disease in Adulthood.. N Engl J Med.

[43] Daniels SR, Arnett DK, Eckel RH, Gidding SS, Hayman LL (2005). Overweight in Children and Adolescents: Pathophysiology, Consequences, Prevention, and Treatment.. Circulation.

[44] Vickers MH, Gluckman PD, Coveny AH, Hofman PL, Cutfield WS (2008). The effect of neonatal leptin treatment on postnatal weight gain in male rats is dependent on maternal nutritional status during pregnancy.. Endocrinology.

