# Supplementary material for: Prenatal Stress Exposure Related to Maternal Bereavement and Risk of Childhood Overweight
Source: PLoS One. 2010 Jul 30;5(7):e11896. doi: 10.1371/journal.pone.0011896 (PMC2912844; doi:10.1371/journal.pone.0011896)
Supplement: Table S1 — Baseline characteristics of the study population.* (0.10 MB DOC) [file pone.0011896.s001.doc]

**Table S1. Baseline Characteristics of the Study Population***

| **Variables** | **Unexposed Cohort** | **Exposed Cohort** | | | |
| --- | --- | --- | --- | --- | --- |
|  |  | **All exposed period** | ***12-7 months before pregnancy*** | ***6-0 months before pregnancy*** | ***During pregnancy*** |
|  | N (%) | N (%) | N (%) | N (%) | N (%) |
|  | 64,753 (100) | 459 (100) | 128 (100) | 170 (100) | 161 (100) |
| **Gender** |  |  |  |  |  |
| Boys | 32,167 (51) | 230 (50) | 64 (50) | 92 (54) | 74 (46) |
| Girls | 31,586 (49) | 229 (50) | 64 (50) | 78 (46) | 87 (54) |
| **Gestational age (weeks)** |  |  |  |  |  |
| <37 | 2,349 (4) | 35 (8) | 12 (8) | 11 (6) | 12 (7) |
| **≥**37 | 40,848 (63) | 386 (84) | 106 (83) | 146 (86) | 134 (83) |
| Unknown | 21,556 (33) | 38 (8) | 10(8) | 13 (8) | 15 (9) |
| **Birth year** |  |  |  |  |  |
| 1970-1978 | 30,431 (47) | 95 (21) | 19 (15) | 52 (31) | 24 (15) |
| 1979-1983 | 14,235 (22) | 118(26) | 40 (31) | 40 (24) | 38 (24) |
| 1984-1989 | 20,087 (31) | 246 (54) | 69 (54) | 78 (46) | 99 (61) |
| **Birth Weight (g)** |  |  |  |  |  |
| <3000 | 12,620 (20) | 107 (23) | 27 (21) | 34 (20) | 46 (29) |
| 3000-3350 | 14,687 (23) | 136 (30) | 37 (29) | 51 (30) | 48 (29) |
| 3350-3700 | 14,515 (22) | 94 (20) | 34 (27) | 34 (20) | 26 (16) |
| >3700 | 15,237 (24) | 108 (24) | 29 (23) | 44 (26) | 35 (22) |
| Unknown | 7,687 (12) | 14 (3) | 1(1) | 7(4) | 6 (4) |
| **Maternal age (years)** |  |  |  |  |  |
| ≤23 | 16,026 (25) | 115 (25) | 26 (20) | 43 (25) | 46 (29) |
| 23-26 | 16,056 (25) | 120 (26) | 35 (27) | 39 (23) | 46 (29) |
| 27-30 | 16,056 (25) | 120 (26) | 31 (24) | 49 (29) | 40 (25) |
| >30 | 16,053 (25) | 104 (23) | 36 (28) | 39 (23) | 29 (18) |
|  |  |  |  |  |  |
| **Maternal education^†^** |  |  |  |  |  |
| Primary | 16,710 (56) | 199 (55) | 59 (55) | 60 (51) | 80 (59) |
| Secondary | 5914 (20) | 81 (23) | 21 (20) | 34 (29) | 26 (19) |
| High | 7286 (24) | 80 (22) | 27 (25) | 24 (20) | 29 (21) |
| **Maternal income^†^** |  |  |  |  |  |
| 1^st^ quartile | 7518 (25) | 85 (24) | 25 (23) | 27 (23) | 33 (24) |
| 2^nd^ quartile | 9384 (31) | 118 (33) | 33 (31) | 41 (35) | 44 (33) |
| 3^rd^ quartile | 9627 (32) | 128 (36) | 40 (38) | 40 (34) | 48 (36) |
| 4^th^ quartile | 3380 (11) | 29 (8) | 9 (8) | 10 (8) | 10 (7) |
| **Maternal cohabitation status^†^** |  |  |  |  |  |
| Single | 11,487 (38) | 168 (47) | 45 (42) | 52 (44) | 71 (52) |
| Cohabitation | 18,483 (62) | 192 (53) | 62 (58) | 66 (56) | 64 (47) |

***values at n (%).**

†Data available only for children born from 1980 to 1989.
